# Supplementary material for: Accurate Detection of Urothelial Bladder Cancer Using Targeted Deep Sequencing of Urine DNA
Source: Cancers (Basel). 2023 May 22;15(10):2868. doi: 10.3390/cancers15102868 (PMC10216686; doi:10.3390/cancers15102868)
Supplement: Supplementary file 1 [file cancers-15-02868-s001.zip › cancers-2342136-Supplementary Figures.pdf]

Accurate Detection of Urothelial Bladder Cancer Using Targeted Deep Sequencing of Urine DNA

Dongin Lee [1], Wookjae Lee [2], Hwang-Phill Kim [2], Myong Kim [3], Hyun Kyu Ahn [3], Duhee Bang [1,*] and Kwang Hyun Kim [3,*]

1 Department of Chemistry, Yonsei University, Seoul 03722, Republic of Korea; egun1229@gmail.com
2 IMBdx, Seoul 08506, Republic of Korea
3 Department of Urology, Ewha Womans University Seoul Hospital, Seoul 07804, Republic of Korea
* Correspondence: duheebang@yonsei.ac.kr (D.B.); khkim.uro@gmail.com (K.H.K.); Tel.: +82-2-2123-2633 (D.B.); +82-2-6986-1685 (K.H.K.); Fax: +82-2-364-7050 (D.B.)

Supplementary Figures

Figure S1

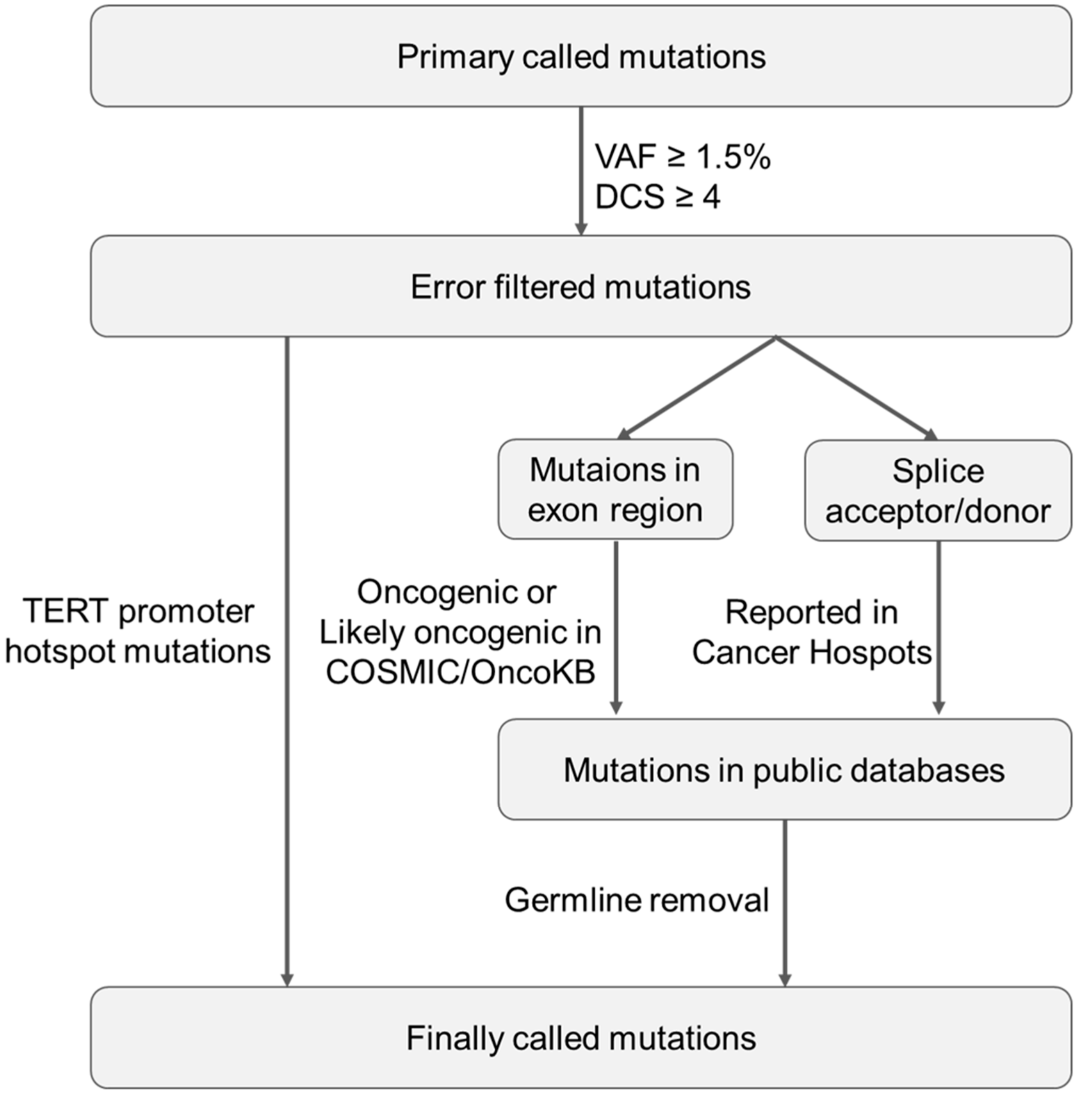

Figure S1 Workflow of mutation calling for utDNA detection without tumor samples.

Figure S2

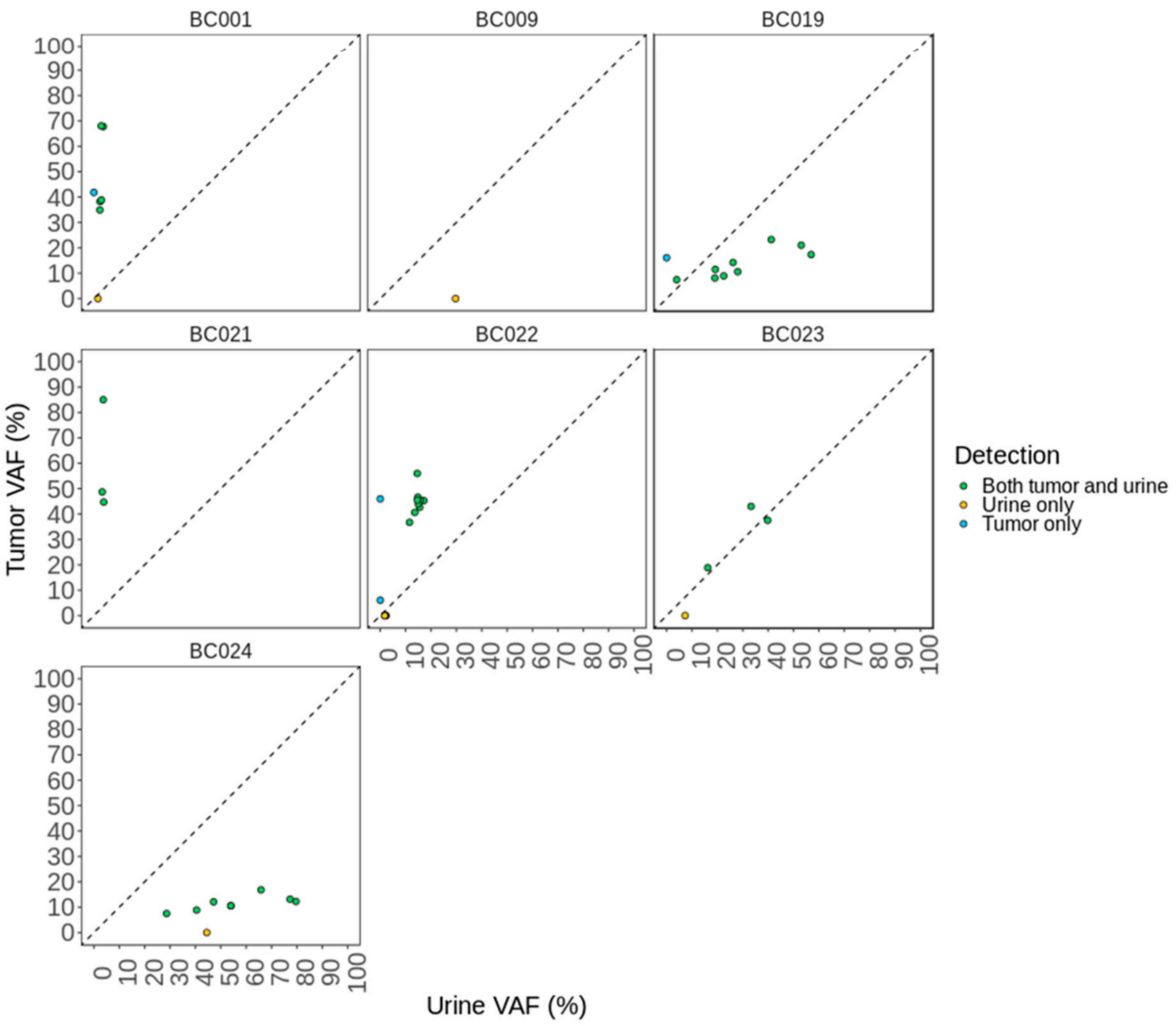

Figure S2 Variant allele frequency (VAF) levels of mutations in seven pairs of tumor and urine samples. Each mutation identified in tumor or urine samples is plotted by its VAFs in paired urine (x-axis) and tumor (y-axis) samples.

Figure S3

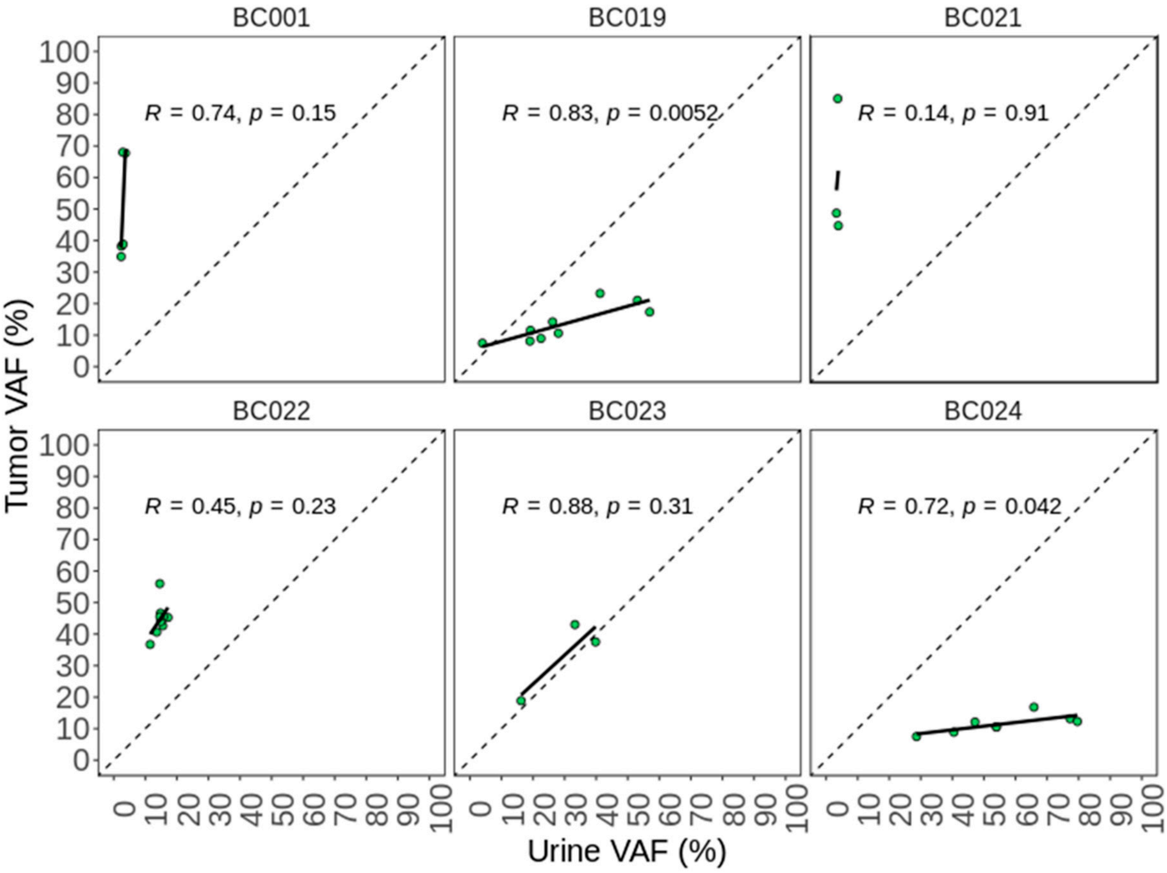

Figure S3 Correlations between VAFs in paired urine and tumor samples. The correlation coefficient (*R*) corresponds to the Pearson correlation coefficient.

Figure S4

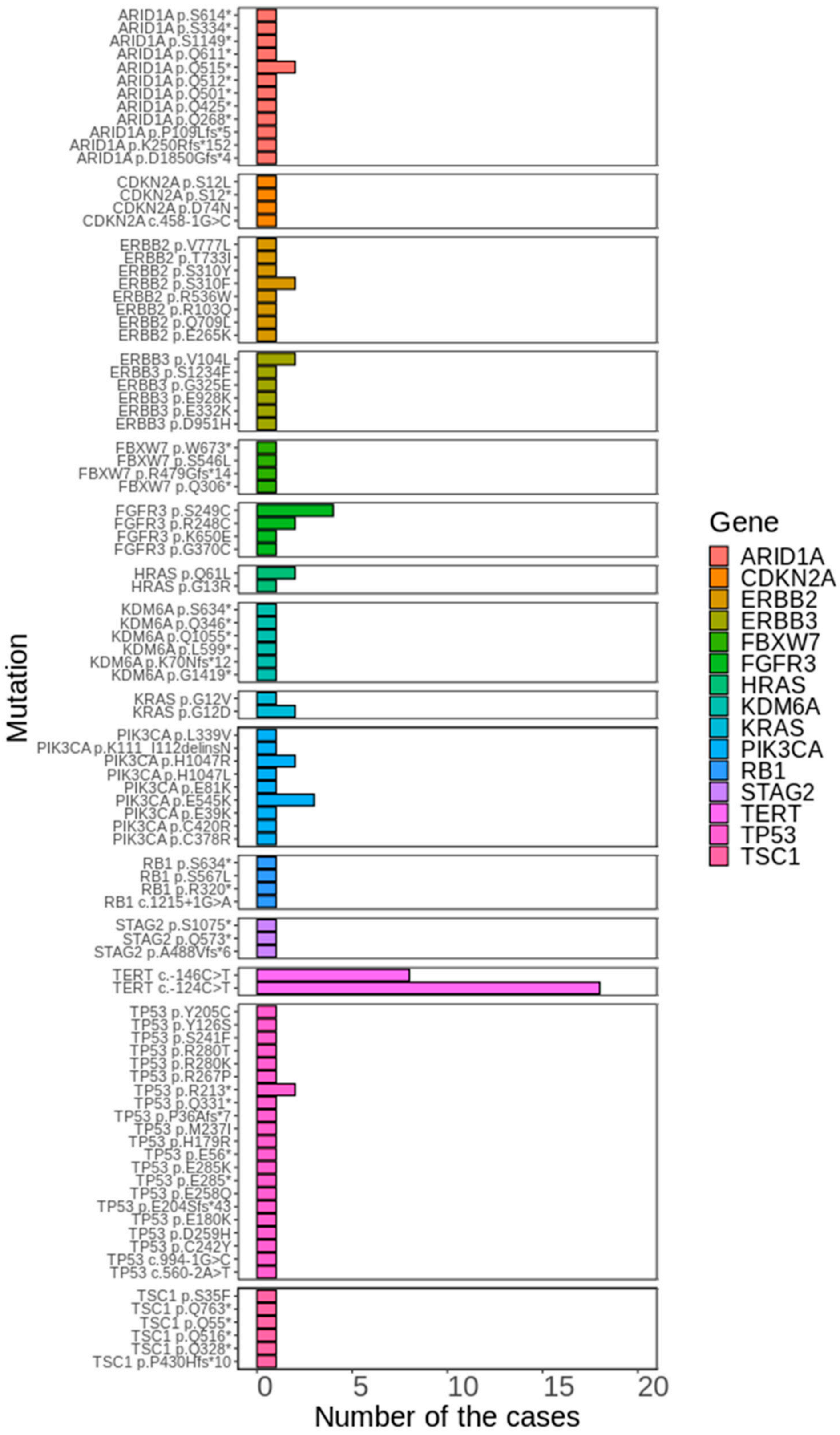

Figure S4 Single-nucleotide variants and insertions/deletions in the 15 most frequently mutated genes. The x-axis denotes the number of cases (BC patients) in whom each mutation was detected in urine samples.

Figure S5

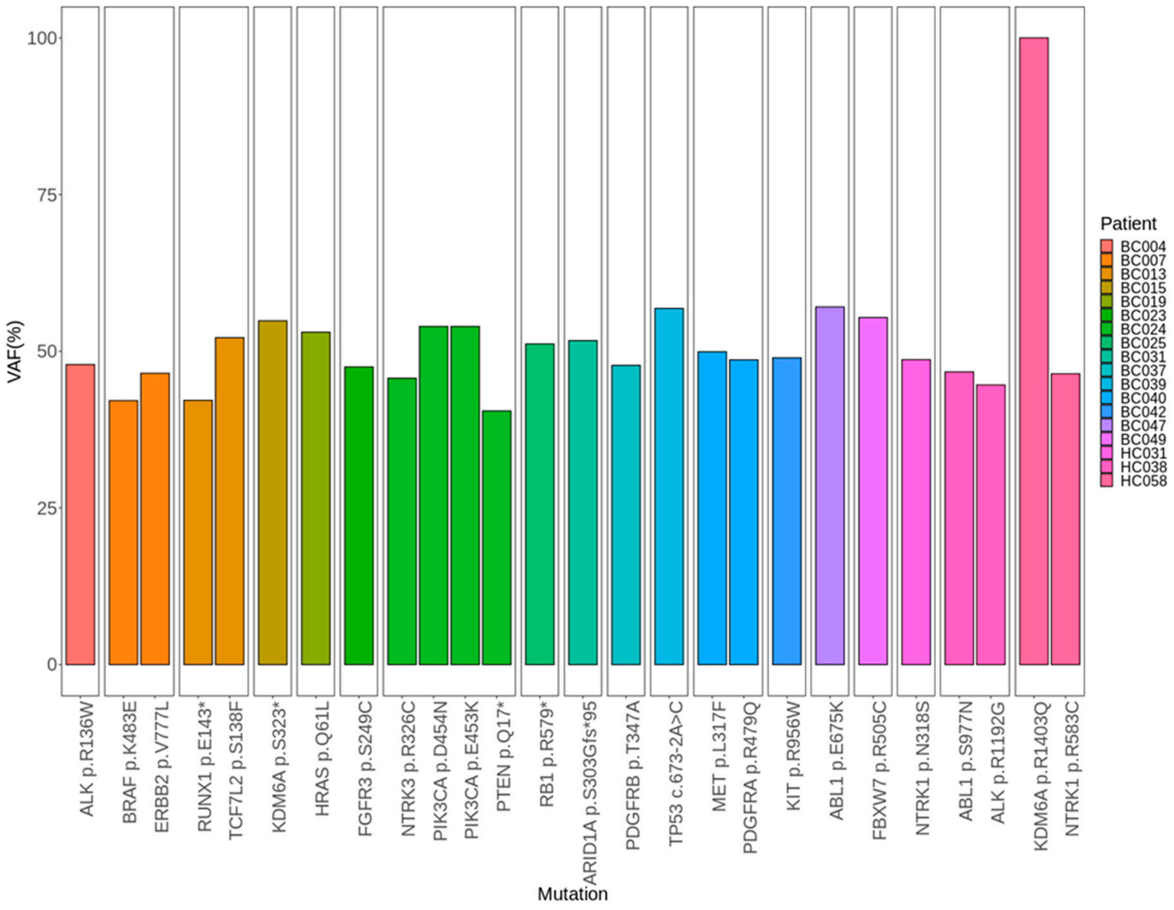

Figure S5 Single-nucleotide variants and insertions/deletions with VAFs in the range of 40~60% or higher than 95%. Colors denote different individuals.

Figure S6

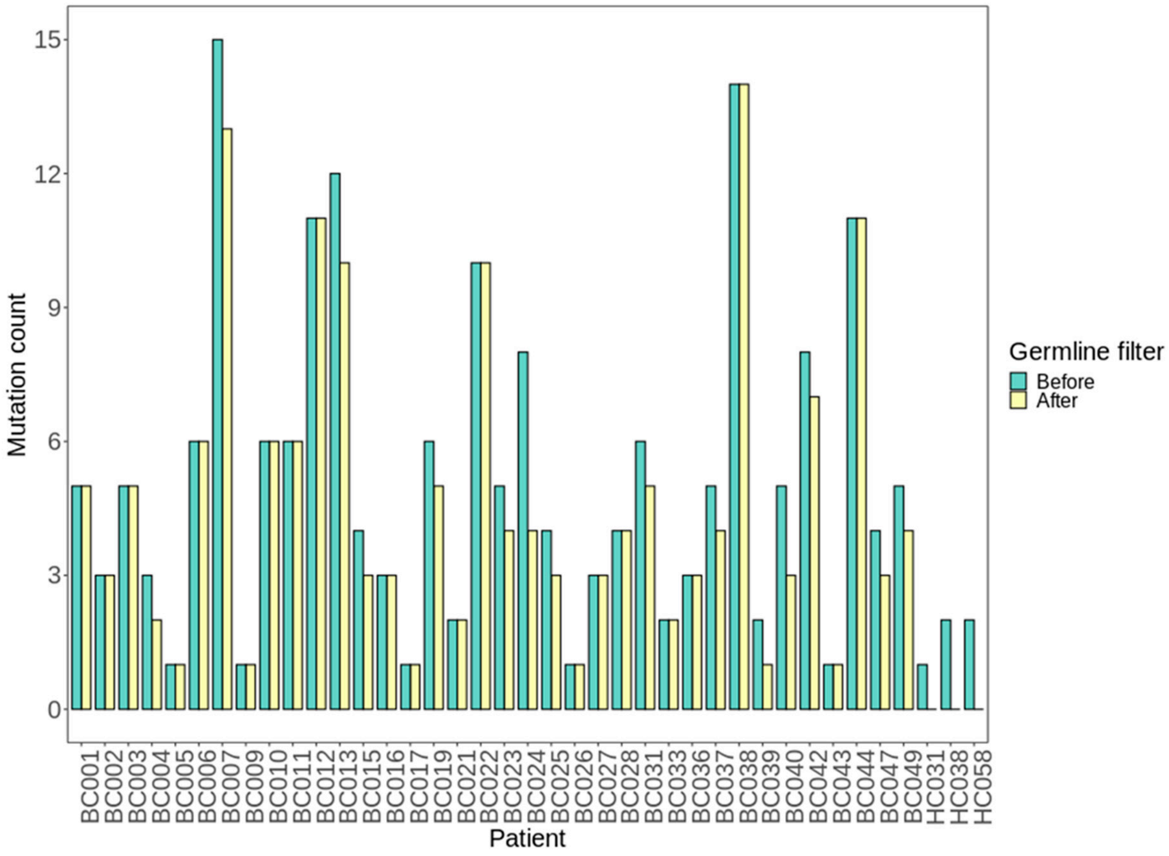

Figure S6 Change in the number of mutations per patient when a germline filter was applied. The green bars denote the number of mutations before applying the germline filter, and the yellow bars denote the number of mutations after applying the germline filter.
